# Assessment of the Diagnostic Agreement Between Cone-Beam Computed Tomography and Intrasurgical Measurements for Detecting Grade II Buccal Furcation Involvement in Maxillary First Molars: Insights on Accuracy, Reliability, and Clinical Relevance

**DOI:** 10.7759/cureus.87515

**Published:** 2025-07-08

**Authors:** Khyati Arora, Harikumar Kanakkath, Nileena R Kumar, Smitha P Sankunni, Rosamma Joseph Vadakkekuttikal

**Affiliations:** 1 Periodontics, Government Dental College, Kozhikode, Kerala University of Health Sciences, Thrissur, IND; 2 Oral Medicine and Radiology, Government Dental College, Kozhikode, Kerala University of Health Sciences, Thrissur, IND

**Keywords:** agreement, clinical significance, cone-beam computed tomography (cbct), furcation, intrasurgical, vernier caliper

## Abstract

Background: The intricate anatomy and challenging accessibility of furcation involvement often complicate diagnosis, leading to tooth loss. This study evaluated the efficacy of cone-beam computed tomography (CBCT) for the early detection and management of furcation defects, particularly Grade II buccal furcations of maxillary first molars. CBCT measurements of width, depth, and height were compared with direct intrasurgical measurements to determine diagnostic accuracy.

Methods: Thirty patients with 41 maxillary first molars with Grade II buccal furcations were clinically diagnosed using Nabers' probe and intraoral periapical radiographs. CBCT scans were performed before surgery, recording measurements for furcation morphology. Intrasurgical measurements were obtained using endodontic files and a digital Vernier caliper.

Results and discussion: CBCT measurements for all parameters were consistently lower than intrasurgical measurements, with statistically significant differences (p < 0.001). Most deviations ranged between -0.50 and 0.50 mm, indicating minimal clinical discrepancies. CBCT demonstrated excellent reliability for depth and height assessments, good reliability for width, and sensitivity in detecting furcation dimensions under 3 mm.

Conclusion: CBCT and intrasurgical measurements for furcation width, depth, and height differ significantly but remain clinically irrelevant (<1 mm). Strong agreement and correlation between CBCT and intrasurgical measurements support its use in pre-treatment diagnosis to improve surgical outcomes. Low-Field-of-view (FOV) CBCT reduces radiation exposure while enhancing image quality, making it ideal for diagnosing furcation defects. Despite differences between CBCT and intrasurgical values, CBCT proves to be a reliable diagnostic tool for predicting furcation morphology.

## Introduction

Periodontitis is a chronic, multifactorial inflammatory disease associated with dysbiotic plaque biofilms and characterized by progressive destruction of the tooth-supporting apparatus [1]. As the disease progresses, it can cause apical migration of the periodontal tissues, exposing the furcation areas of multirooted teeth. This exposure results in irreversible interradicular bone loss, leading to tooth loss if left untreated. Furcation defects manifest as three-dimensional (3D) lesions in the interradicular bone and can be measured in terms of width, depth, and height. Furcation involvement is more prevalent in maxillary molars compared to mandibular molars, with the highest incidence occurring in maxillary first molars, followed by maxillary second molars, mandibular first molars, and mandibular second molars [2]. Among these, Grade II furcation involvement is the most common, and the buccal furcation of the maxillary molar is particularly susceptible [3]. According to the Glickman grading, Grade II furcation involvement is defined as loss of interradicular bone and pocket formation, but a portion of the alveolar bone and periodontal ligament remains intact [4]. Teeth affected by furcation involvement have a notably poor prognosis, as evidenced by studies reporting significantly higher rates of tooth loss in furcation-involved teeth compared to single-rooted teeth. Hirschfeld and Wasserman observed a 31.4% tooth loss in multirooted teeth with furcation involvement over a 22-year maintenance period, whereas single-rooted teeth exhibited a much lower rate of 4.9% [5]. Similarly, McFall [6] reported a 57% loss in teeth with furcation involvement, highlighting the critical need for accurate diagnosis and intervention.

The diagnosis of furcation involvement relies on clinical examination and radiographic imaging. However, due to the complex anatomy of maxillary furcation areas, appropriate diagnosis is often challenging. Conventional two-dimensional (2D) radiographs provide limited information, making them inadequate for precise pretreatment assessment of furcation defects [7]. Cone-beam computed tomography (CBCT), introduced in periodontal diagnostics by Misch et al. [8], offers a 3D imaging alternative, reconstructing dental anatomy in axial, coronal, and sagittal planes. This enables a more detailed evaluation of furcation defects, potentially enhancing diagnostic accuracy [9]. Despite its advantages, CBCT may still underestimate or overestimate the true extent of furcation involvement when compared to direct surgical assessment [10,11].

Direct intrasurgical measurement remains the gold standard for assessing furcation defects, as it provides the most precise detection of bone loss and enables the evaluation of regenerative outcomes [11]. Nonetheless, this method is limited to intraoperative settings, making preoperative planning difficult. A reliable correlation between CBCT and intrasurgical measurements could make CBCT a valuable tool for pretreatment diagnosis, aiding in better planning for appropriate treatment and thereby improving the prognosis of furcation-involved teeth. This study aimed to assess the sensitivity and specificity of CBCT in diagnosing Grade II buccal furcation involvement and evaluate the agreement and correlation between CBCT measurements and direct intrasurgical measurements.

## Materials and methods

Study design

This study has a comparative observational design.

Study setting

This study was conducted in the Department of Periodontics, Govt Dental College, Kozhikode, and the Department of Oral Medicine and Radiology, Govt Dental College, Kozhikode.

Study duration 

This study commenced on May 5, 2022, and was completed on February 1, 2024 (21 months). Ethical clearance was obtained from the Institutional Ethics Committee, Govt Dental College, Kozhikode, IEC No. 238/2022/DCC dated May 4, 2022.

Sample size calculation

The sample size was estimated using the formula n = (Zα + Zβ)^2^ × SD^2^/d^2^, where n = sample size, SD = standard deviation, d = difference in mean, Zα = 1.96, Zβ = 0.84, SD = 1.12, and d = 0.5.

n = (1.96 + 0.84)^2^ × 1.12^2^/(0.5)^2^ = 40

Forty-one furcation sites were evaluated. A total of 41 furcation sites in 30 patients were selected from the Department of Periodontics, Govt Dental College, Kozhikode, with Grade II buccal furcation defect of maxillary first molar according to Glickman grading [4].

Inclusion and exclusion criteria

Thirty patients with 41 maxillary molars with Grade II buccal furcations were included in this study as per the inclusion and exclusion criteria mentioned below. Patients aged between 20 and 60 years reporting to the Department of Periodontics, Govt Dental College, Kozhikode, Kerala, and diagnosed with Grade II buccal furcation involvement in the maxillary first molar by clinical measures as per the Glickman classification system were included in the study [4].

Patients with systemic diseases, smokers, pregnant subjects, patients with caries, and metallic restorations in the furcation were excluded from the study. One buccal furcation was considered as one statistical unit. All patients signed informed consent before participating in the study. We used the Standards for Reporting Diagnostic Accuracy (STARD) [12] reporting guideline to draft this manuscript and the STARD reporting checklist when editing [13].

Study procedure

Study subjects were interviewed using a detailed questionnaire. A straight periodontal probe with William’s graduation and Nabers' probe with marking at every 3 mm were used to measure PPD, CAL, the vertical component, and the horizontal component of furcation. Patients diagnosed with Grade II buccal furcation as per the inclusion criteria were referred to the Department of Oral Medicine and Radiology. CBCT imaging of the selected maxillary first molars was carried out, and the radiographic parameters were recorded.

Radiological parameters

A CBCT scan was done using the Planmeca ProMax 3D MiD extraoral imaging system (Planmeca, Helsinki, Finland) with a flat panel detector (90 kV, 8 mA, 12 s, voxel size of 200 µm, field of view (FOV) 4 × 5 cm, DAP 333 mGy/cm^2^); multiplanar reformatted images were obtained and evaluated independently by a trained radiologist, in a darkened windowless room, on a 24-inch thin film transistor (TFT) screen. The DICOM files were analyzed on a PC workstation with Microsoft Windows 10 (Microsoft Corp., Redmond, WA, US) using the software Planmeca Romexis. The furcation entrance served as an anatomical starting point for measurements. CBCT was analyzed in axial, sagittal, and coronal sections, which made the defect most visible and easily measurable. The cross-sections of different planes were aligned using a furcation entrance as an anatomical landmark. Width, height, and depth measurements were performed by measuring the deepest vertical and horizontal furcation defect at each furcation entrance. Back-and-forth scrolling in different planes allowed us to identify and measure the deepest vertical and horizontal extent of bone loss. Radiographic parameters were recorded by a trained examiner. A pilot study was conducted on 10 furcation sites, and radiographic measurements were obtained by three trained examiners. Intraclass correlation (ICC) was calculated, which showed excellent correlation (ICC = 0.91) between the three measurements.

Intrasurgical measurements

The conventional flap was reflected under local anesthesia. Debridement of the periodontal defect in the furcation area was done thoroughly. Direct surgical measurements (width, depth, and height) were made using the endodontic file and were transferred to a digital Vernier caliper to obtain maximum accuracy. All intrasurgical measurements were done by the same trained assessor in all sites as per the following criteria, and calibration of the Vernier caliper was done before the study. A pilot study was conducted on 10 furcation sites, and measurements were obtained by three trained examiners. ICC was calculated, which showed excellent correlation (ICC = 0.94) between the three measurements.

**Figure 1:**
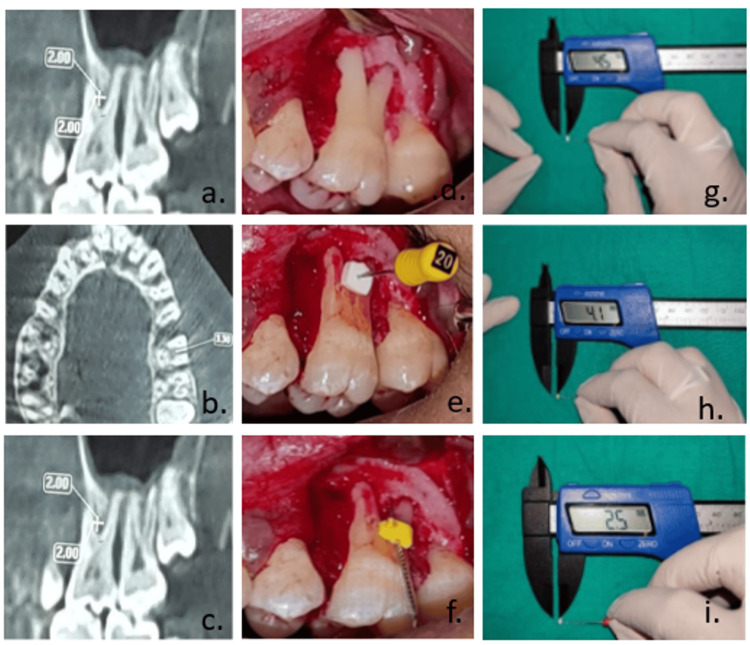
Measurement process using CBCT and Vernier caliper. (a-c) CBCT measurements of width, depth, and height, respectively. (d-i) Intrasurgical measurements transferred to Vernier caliper for precise assessment of width, depth, and height CBCT: cone-beam computed tomography

Height was measured from the furcation fornix to the base of the alveolar bone. Width was measured between the greatest dimensions of separation between the two roots above the crest of the alveolar bone.·Depth was measured from the crest of the alveolar bone till the interradicular bony resistance was felt (Figure 1).

Statistical analysis

Data management and analysis were done using SPSS Version 22 (IBM Corp., Armonk, NY, US). The significance level (α) was set at 5%, with a power (1-β) of 80%. Mean ± standard deviation (SD) was used for continuous variables, and proportion for qualitative variables. Normality was analyzed by the Shapiro-Wilk test. Data were not normally distributed, so a non-parametric test (Wilcoxon signed-rank test) was used for analyzing the difference between CBCT and intrasurgical measurements. ICC was done between CBCT and intrasurgical measurements. A scatter plot was used to assess the agreement between CBCT and intrasurgical measurement. Sensitivity and specificity were calculated for CBCT measurements (depth and height) in diagnosing furcation in early stages.

## Results

The study comprised 30 participants, out of whom 19 were men and 11 were women. In 30 participants, 41 Grade II maxillary buccal furcations were assessed in terms of width, depth, and height. Table 1 shows the demographic status of study participants. Table 2 shows the mean ± SD and median ± interquartile range (IQR) for CBCT and intrasurgical measurement in measuring the width, depth, and height of the furcation. The Shapiro-Wilk test was done to assess the normality distribution of parameters. The mean of CBCT and intrasurgical measurement in terms of width was 1.95 ± 0.82 and 2.24 ± 0.76, respectively. The mean of CBCT and intrasurgical measurement in terms of depth was 3.27 ± 0.76 and 3.57 ± 0.76, respectively. The mean of CBCT and intrasurgical measurements in terms of height was 2.96 ± 2.14 and 3.24 ± 2.17, respectively. The above findings suggest that the means of CBCT measurements for width, depth, and height were less than those of intrasurgical measurements. Table 3 shows the test of significance for the difference in the median of CBCT and intrasurgical measurements in terms of width, depth, and height using the related sample Wilcoxon signed-rank test. Wilcoxon signed-rank test statistics (W) for width, depth, and height was 823, 821, and 846, respectively, which suggest that there was a statistically significant difference between CBCT and intrasurgical measurements (p < 0.001).

**Table 1:** Demographics of study participants N: frequency; %: percentage; APL: above poverty line; BPL: below poverty line

Demographic variables		N (%)
Gender frequency N (%)	Males	19 (63.33)
	Females	11 (36.66)
Religion frequency N (%)	Hindu	24 (80)
	Muslim	6 (20)
	Christian	0
Education frequency N (%)	Primary school	7 (23.3)
	Middle school	9 (30.0)
	High school	10 (33.3)
	Diploma	1 (3.3)
	Graduate	2 (6.7)
	Professional	1 (3.3)
Occupation frequency N (%)	Unemployed	8 (26.7)
	Unskilled worker	5 (16.7)
	Semiskilled worker	12 (40.0)
	Skilled worker	1 (3.3)
	Clerical/shop owner/farmer	1 (3.3)
	Professional	1 (3.3)
	Business	1 (3.3)
	Student	1 (3.3)
Socioeconomic status frequency N (%)	APL	16 (53.3)
	BPL	14 (46.7)

**Table 2:** Mean ± SD and median ± IQR for CBCT and intrasurgical measurements in measuring width, depth, and height of furcation (in mm) SD: standard deviation; IQR: interquartile range; CBCT: cone-beam computed tomography; mm: millimeters

Measurements	Mean ± SD	Median ± IQR
CBCT width	1.95 ± 0.82	1.80 ± 0.80
Intrasurgical width	2.24 ± 0.76	2.00 ± 0.80
CBCT depth	3.27 ± 0.76	3.11 ± 2.60
Intrasurgical depth	3.57 ± 0.76	3.50 ± 2.70
CBCT height	2.96 ± 2.14	2.30 ± 1.46
Intrasurgical height	3.24 ± 2.17	2.40 ± 1.95

**Table 3:** Comparison of CBCT and intrasurgical measurements using Wilcoxon signed-rank test in terms of width, depth, and height (in mm) Q1: lower quartile; Q3: upper quartile; W: Wilcoxon signed-rank test statistics; CBCT: cone-beam computed tomography *p < 0.05 is considered statistically significant

Measurements (mm)	CBCT median (Q1, Q3)	Intrasurgical median (Q1, Q3)	Test statistics (W)	p-value
Width	1.80 (1.41, 2.20)	2.00 (1.80, 2.60)	823	<0.001*
Depth	3.11 (1.76, 4.36)	3.50 (2.00, 4.70)	821	<0.001*
Height	2.30 (1.80, 3.26)	2.40 (2.00, 3.95)	846	<0.001*

**Figure 2:**
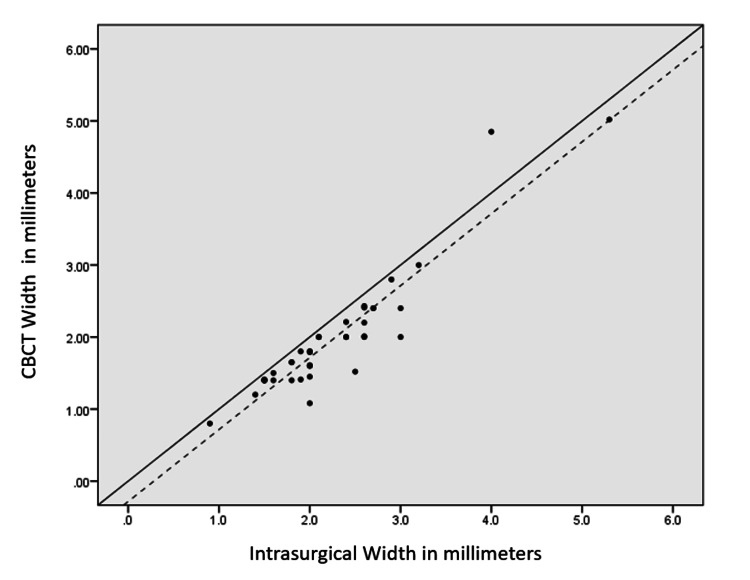
Scatter plot showing a correlation between intrasurgical and CBCT measurements (in mm) in terms of width The dotted line is the trend line or line of best fit through the observed values plotted using least squares estimation [15]. The trend line is near the line of perfect agreement with an intraclass correlation coefficient of 0.873, showing there is agreement as well as correlation between the two measurements CBCT: cone-beam computed tomography

**Figure 3:**
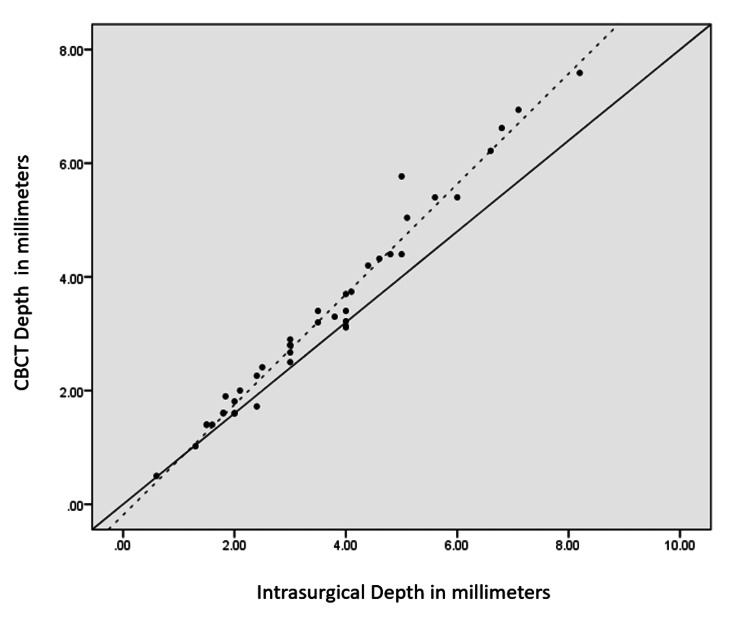
Scatter plot showing a correlation between intrasurgical and CBCT measurements (in mm) in terms of depth The trend line is near the line of perfect agreement with an intraclass correlation of 0.973, showing there is correlation as well as agreement between the two measurements CBCT: cone-beam computed tomography

**Figure 4:**
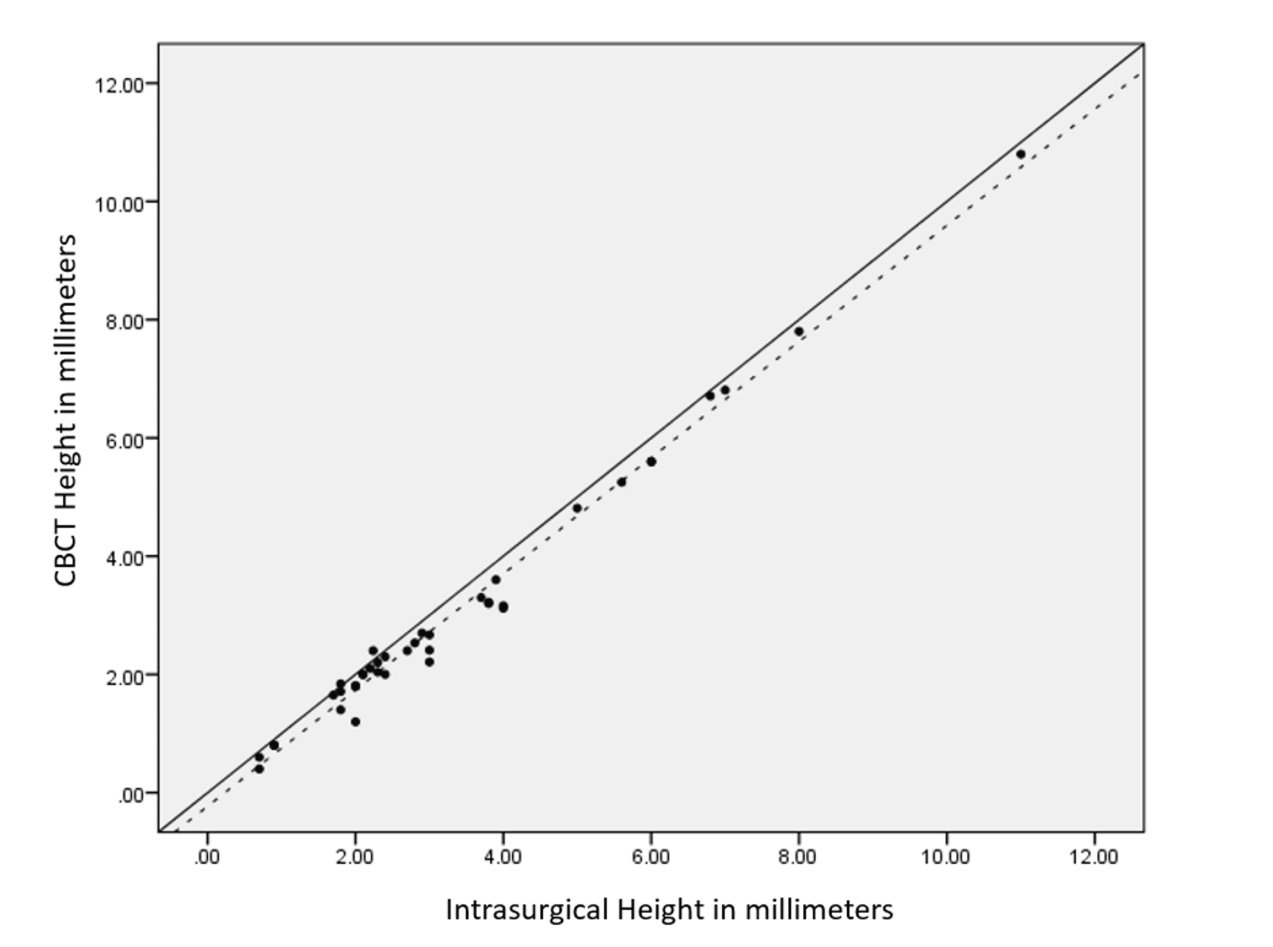
Scatter plot showing a correlation between intrasurgical and CBCT measurement (in mm) in terms of height The trend line is near the line of perfect agreement with an intraclass correlation of 0.985, showing correlation and agreement between the two measurements In all the scatter plots, the trend line is below and close to the line of perfect agreement (solid black line), suggesting CBCT underestimates the furcation measurements in terms of width, depth, and height CBCT: cone-beam computed tomography

For a more in-depth analysis, comparison frequency distribution of patients based on the deviation between CBCT and intrasurgical measurements for width, depth, and height was calculated, as shown in Table 4. From Table 4, the following are observed: for width, within 97.6% of the furcation sites, CBCT showed lesser values than intrasurgical measurement, with 80.5% showing a difference of -0.50 to 0.50 mm and only 19.5% showing a difference of >0.5 mm between CBCT and intrasurgical measurements; for depth, within 95.2% of the furcation sites, CBCT showed lesser measurements than intrasurgical measurement, with 82.9% showing a difference of -0.50 to 0.50 mm and 17.1% showing a difference of >0.5 mm between CBCT and intrasurgical measurements; and for height, within 95.2% of the furcation sites, CBCT showed lesser values than intrasurgical measurement, with 78.9% showing a difference of -0.50 to 0.50 mm and 21.9% showing a difference of >0.5 mm between CBCT and intrasurgical measurements. ICC was done between CBCT and intrasurgical measurements for width, depth, and height, as shown in Table 5. The ICC coefficient for width, depth, and height was 0.873, 0.973, and 0.985, respectively, which suggest that CBCT showed good reliability for measuring furcation width and showed excellent reliability for depth and height of furcation (value of 0.5 shows poor reliability, values between 0.5 and 0.75 indicate moderate reliability, values between 0.75 and 0.9 indicate good reliability, and values greater than 0.90 indicate excellent reliability) [14]. There was a correlation as well as an agreement between CBCT and intrasurgical measurements, as shown in Table 5 and Figures 2-4.

**Table 4:** Frequency distribution of the furcation sites based on the deviation between CBCT and intrasurgical measurements (+1 mm to -1 mm) in measuring width, depth, and height N: no. of furcation sites; %: percentage of furcation sites; CBCT: cone-beam computed tomography

Deviation level (-1 mm to +1 mm)	Width N (%)	Height N (%)	Depth N (%)
-1.00 to -0.51	7 (17.1)	7 (17.1)	8 (19.5)
-0.50 to -0.11	22 (53.7)	20 (48.8)	23 (56.1)
-0.10 to -0.01	11 (26.8)	12 (29.3)	8 (19.5)
0	0 (0)	0 (0)	0 (0)
0.01 to 0.10	0 (0)	1 (2.4)	1 (2.4)
0.11 to 0.50	0 (0)	1 (2.4)	0 (0)
0.51 to 1.00	1 (2.4)	0 (0)	1 (2.4)
Total	41 (100)	41 (100)	41 (100)

**Table 5:** Intraclass correlation between CBCT and intrasurgical measurements in terms of width, depth, and height CBCT: cone-beam computed tomography

	Intraclass correlation between CBCT and intrasurgical measurements	95% confidence interval	
		Lower bound	Upper bound
Width	0.873	0.435	0.955
Depth	0.973	0.783	0.992
Height	0.985	0.807	0.996

For better data interpretation, all observations were plotted in a scatter plot. The scatter plot in Figures 2, 3 shows a correlation between intrasurgical and CBCT measurements in terms of width, depth, and height. The dotted line represents the trend line or line of best fit through the observed values plotted using the least squares estimation. Figure 2 (width) shows that the trend line was near the line of perfect agreement with an ICC coefficient of 0.873, showing there was agreement as well as correlation between the two measurements. Figure 3 (depth) shows that the trend line was near the line of perfect agreement with an ICC of 0.973, showing there was correlation as well as agreement between the two measurements. Figure 4 (height) shows that the trend line was near the line of perfect agreement with an ICC of 0.985, showing correlation and agreement. In all the graphs, the trend line was below and close to the line of perfect agreement (solid black line), suggesting CBCT underestimates the furcation measurements in terms of width, depth, and height.

Table 6a suggests that CBCT successfully identified all 21 cases (51.22%) where the intrasurgical depth was ≥3 mm, indicating high sensitivity for detecting severe furcation defects. CBCT identified 15 cases (36.5%) correctly but underestimated five cases (12.1%), where actual surgical findings showed deeper involvement. Interestingly, CBCT never predicted a depth ≥3 mm when intrasurgical measurements were <3 mm, suggesting a tendency toward conservative estimations. Table 6b indicates that CBCT demonstrated strong sensitivity in detecting less furcation involvement height (<3 mm), correctly identifying 80.47% of cases. This indicates that CBCT is effective for preliminary diagnosis, particularly in cases with less furcation height. However, CBCT showed 19.53% accuracy in identifying more furcation height (≥3 mm), indicating a potential underestimation of severe cases. The results demonstrated 100% sensitivity for both depth and height assessment, indicating that CBCT effectively identifies furcation involvement when present. The specificity was 80.76% for depth measurements. However, for height assessment, CBCT showed 100% specificity, correctly classifying all cases without false positives, as shown in Tables 7, 8.

**Table 6:** Comparative analysis of CBCT and intrasurgical measurements (gold standard) for furcation depth (a) and height (b) <3 mm and ≥3 mm in measuring furcation involvement N: no. of furcation sites; %: percentage of furcation sites; CBCT: cone-beam computed tomography

Table 6a		Intrasurgical depth N (%)		Total
		<3	≥3	
CBCT depth	<3	5 (36.5)	5 (12.1)	20 (48.78)
	≥3	0 (0)	21 (51.22)	21 (51.22)
Total		15	26	41 (100)

Table 6b		Intrasurgical height N (%)		Total
		<3	≥3	
CBCT depth	<3	33 (80.47)	0	33 (80.47
	≥3	0 (0)	08 (19.53)	08 (19.53)
Total		31 (75.60)	8 (19.53)	41 (100)

**Table 7:** Comparative analysis of CBCT and intrasurgical measurements < 3 mm and ≥3 to calculate sensitivity and specificity of CBCT measurements in diagnosing Grade II buccal furcation involvement of maxillary first molars before surgery N: no. of furcation sites; %: percentage of furcation sites; CBCT: cone-beam computed tomography

	CBCT depth N (%)	Intrasurgical depth N (%)	CBCT height N (%)	Intrasurgical height N (%)
<3	20 (48.78)	15 (36.58)	33 (80.47)	33 (80.47)
≥3	21 (51.22)	26 (63.41)	8 (19.53)	8 (19.53)

**Table 8:** Sensitivity and specificity of CBCT in measuring furcation depth and height CBCT: cone-beam computed tomography

	Depth	Height
Sensitivity of CBCT	100%	100%
Specificity of CBCT	80.76%	100%

## Discussion

The involvement of furcation in periodontitis significantly complicates treatment strategies. The anatomical characteristics associated with furcation involvement make diagnosis challenging. Denudation of bone within the furcation area results in a 3D defect in the interradicular bone. Maxillary molars exhibiting furcation involvement can be quantified in terms of their width, depth, and height. These parameters can be measured more precisely following surgical exposure of the defect. Hence, intrasurgical measurements are considered more accurate.

Accurate pretreatment diagnosis is essential to improve clinical outcomes in surgical furcation therapy. Ideally, the assessment of the 3D components of furcation in maxillary molars should align with intrasurgical measurements. Preoperative diagnostic accuracy plays a crucial role in predicting prognosis and guiding treatment selection. Clinical and 2D radiographic evaluations, however, are limited in their ability to assess the 3D components of furcation comprehensively. CBCT has been recognized as an effective tool for evaluating 3D furcation parameters; however, some studies indicate that CBCT may either overestimate or underestimate furcation defects [10,11].

This study aimed to compare the agreement between pretreatment CBCT measurements and intrasurgical measurements of width, depth, and height in Grade II buccal furcation of maxillary first molars. The mesial and distal furcations were not included due to the difficulty of measuring their 3D components intrasurgically. Furthermore, the prevalence of buccal furcation involvement in maxillary first molars is higher than that in mesial or distal furcations [2].

To enhance the reliability of data, 3D furcation defect measurements were performed using an endodontic file and stopper, with measurements transferred to a digital Vernier caliper. This method was adapted from a similar technique by Padmanabhan et al. for measuring 3D components of mandibular molars [11]. A study by Misch et al. demonstrated that digital Vernier calipers offer precise measurements when compared to conventional probes for measuring interproximal defects, with statistically significant differences [8].

In this study, the Planmeca ProMax 3D MiD extraoral imaging system was employed, utilizing a small FOV with a slice thickness of 200 µm to optimize spatial resolution and reduce radiation dose. Smaller slice thickness improves accuracy, as supported by prior studies using systems such as 3D Accuitomo (J. Morita Corp., Kyoto, Japan) and NewTom 3G (NewTom, Imola, Italy), which used a slice thickness of 0.5 mm [16]. Smaller FOVs were used to reduce the radiation exposure [17].

The comparison of CBCT and intrasurgical measurements revealed that CBCT measurements consistently underestimated the actual dimensions of the furcation in terms of width, depth, and height, as shown in Table 2. Statistically significant differences were noted across all parameters (Table 3). However, while significant, the mean differences (0.33 mm for width, 0.30 mm for depth, and 0.28 mm for height) were minimal and not clinically meaningful. Frequency distributions further supported this finding, with all discrepancies being less than 1 mm, as shown in Table 4.

The agreement between the two measurement techniques was assessed using ICC and scatter plots (Table 5 and Figures 2, 3). Good agreement was found for furcation width (ICC = 0.873), while excellent correlations were observed for depth (ICC = 0.973) and height (ICC = 0.985) [16]. The scatter plots showed a linear positive correlation, indicating a strong relationship between CBCT and intrasurgical measurements. Although statistically significant differences were observed, the ICC and scatter plot data indicate that the measurements were highly reproducible and reliable.

Additionally, CBCT demonstrated sensitivity in detecting furcations with depths and heights less than 3 mm, emphasizing its utility in early-stage furcation involvement detection, as shown in Tables 6-8. CBCT emerges as a highly sensitive and specific tool for evaluating furcation involvement, particularly height measurements. At the same time, depth measurements show excellent sensitivity. Clinicians should use CBCT findings in conjunction with clinical examination and intrasurgical validation when planning regenerative surgeries, ensuring optimal treatment strategies.

Clinical relevance

Furcation involvement is a significant risk factor for tooth loss. However, diagnosing and measuring furcation defects remain challenging due to limited access, morphological variations, and measurement errors. This study shows that despite statistically significant differences, CBCT measurements are highly correlated with intrasurgical findings and can be considered reliable for diagnosing furcation morphology in maxillary first molars. Although CBCT values tend to be slightly lower than actual measurements, the discrepancy is not clinically meaningful. Presurgical 3D assessment using CBCT can guide treatment planning and improve the outcomes of regenerative furcation therapy, ultimately enhancing the prognosis for furcation-involved teeth.

Limitations of the study

The limitations of the study are the following: (1) The sample size was small; further studies are needed with a larger sample size to confirm the agreement between the two measurements. (2) Due to access difficulty while taking intrasurgical measurements, the mesial and distal furcations were not included in this study. (3) Sensitivity and specificity were calculated for depth and height but not for width. To the best of our knowledge, no classification to date numerically classifies furcation based on the width.

## Conclusions

Within the limits of this study, CBCT proves to be a reliable diagnostic tool for assessing furcation involvement, despite its slight underestimation compared to intrasurgical measurements. The discrepancy, being clinically insignificant (<1 mm), does not compromise its practical utility. Strong ICC and scatter plot analysis confirm good agreement between CBCT and surgical measurements, supporting its role in pretreatment diagnosis for improved surgical outcomes. Additionally, low FOV CBCT is recommended as it enhances image contrast while minimizing radiation exposure. Overall, CBCT serves as a valuable tool in prognosis assessment, treatment planning, and post-treatment evaluation of furcation defects.
